# Infective Endocarditis Hospitalizations in Kentucky, 2008–2018: Spatial and Temporal Trends

**DOI:** 10.13023/jah.0801.03

**Published:** 2026-04-01

**Authors:** Meera Marji, Steve Browning, W. Jay Christian, Richard Charnigo, Steve W. Leung, Anna Kucharska-Newton

**Affiliations:** University of Kentucky; University of Kentucky; University of Kentucky; University of Kentucky

**Keywords:** Appalachia, geospatial clusters, infective endocarditis, opioid epidemic

## Abstract

**Introduction:**

Infective endocarditis (IE) hospitalization rates have increased significantly in the last decade, primarily due to the opioid epidemic. In 2016, Kentucky (KY) ranked among the top ten states for opioid-related overdose deaths, but limited data exist on IE trends in KY.

**Purpose:**

This study seeks to examine county-level spatial and temporal trends in IE hospitalization rates in KY.

**Methods:**

Hospital inpatient discharge data from 2008–2018 for KY residents aged 18 and older were analyzed. IE admissions and drug use status were identified using ICD-9 & 10 diagnosis codes. U.S. Census data calculated yearly IE hospitalization rates per 100,000 residents. Joinpoint regression identified periods with significant rate changes and calculated annual percent change (APC). Poisson modeling in SaTScan conducted spatiotemporal cluster analysis at the county level and calculated the relative risk (RR) of IE hospitalizations within identified clusters.

**Results:**

During 2008–2018, we observed 17,787 IE hospital admissions, including 3,577 associated with intravenous drug use (IDU-IE). Medicaid patients were more prevalent in the IDU-IE group compared to the non-IDU-IE group (75% vs. 15%). IDU-IE hospitalization rates increased from 1.2 to 27.3 per 100,000 residents between 2008 – 2018. Joinpoint regression for IDU-IE identified a significant upward trend from 2013–2018 with an APC of 71.3% (95% CI: 47.8, 98.5). The spatiotemporal analysis identified seven significant IE hospitalization clusters, with the largest (RR=2.06, p

**Implications:**

IDU-IE hospitalization rates increased significantly from 2013–2018, while non-IDU-IE rates decreased over the study period.

## INTRODUCTION

Infective endocarditis (IE) is a life-threatening microbial infection involving the heart’s valves.^1^ In the United States (U.S.) general population, this condition is rare, with an annual incidence ranging from 3.6 to 7.0 per 100,000 persons.^2^ In the U.S., endocarditis hospitalization rates have increased significantly in the last decade, largely due to an epidemic in intravenous (mis)use of prescription opioid drugs.^3^,^4^

A North Carolina (NC) study reported an increase in hospitalization rates for intravenous drug use-associated infective endocarditis (IDU-IE) from 0.92 per 100,000 persons in 2007 to 10.95 per 100,000 persons in 2017,^3^ with the cost of care for IE hospitalizations increasing from $1.1 million in 2010 to $22.2 million in 2015.^5^ IDU-IE is potentially fatal, with estimates of in-hospital mortality ranging from 5% to 20%^2^,^6^ and up to 50% when surgery is needed but not performed 7.

The prevalence of IE correlates strongly with the prevalence of drug use. There are several reasons that people who inject drugs (PWID) are at increased risk of developing IE. First, injected particulate matters containing talcum and bacteria that get introduced into the circulatory system through inexpert injections and repeat needle use lead to endothelial tissue damage, resulting in subsequent damage to valves of the heart.^2^,^8^ Although much recent attention has focused on the rise in IE associated with prescription opioid injection, other injected substances, including cocaine, can also contribute to risk. The active substance in cocaine may directly harm cardiac tissue and lead to valve damage via physiologic vasospasms, which make the area more susceptible to infections.^2^

Numerous data sources document a high prevalence of opioid use in Kentucky (KY). In 2016, the state ranked among the top ten states with the highest opioid-related overdose deaths.^9^ In late 2017, the Northern Kentucky Health Department reported a significant rise in injection drug use, especially among those diagnosed with human immunodeficiency virus (HIV).^10^ In 2015, the Appalachian part of KY was reported to have the highest burden of drug overdose among the 13 U.S. Appalachian states, along with West Virginia and Appalachian Ohio.^11^ Moreover, the Appalachian KY region showed a 30% higher rate of opioid prescriptions per 1,000 population than central KY.^12^

Trends of IE among drug users have been studied nationally. State-specific studies are available for only a few states, and very limited data regarding IE prevalence are available for KY. This article describes county-level hospitalization rates and trends of IE in KY between 2008 – 2018.

## METHODS

### Study Population

Hospital inpatient discharge data from 2008 – 2018 were provided by the KY Cabinet for Health and Family Services (CHFS) through the University of Kentucky Center for Clinical and Translational Science (CCTS). The database includes information on all annual inpatient discharge data from 130 hospitals in KY (>98% of hospitals). Hospitalizations for KY residents 18 years and older were included in the analysis. Admissions from border states were excluded.

### Assessment of IE Admissions and Drug Use Status

All IE admissions were identified using the International Classification of Diseases (ICD), Ninth and Tenth Revisions, Clinical Modification codes (ICD-9 CM- 421.0, 421.1, 421.9, 424.90, 424.91, 424.99, 112.81 and ICD-10 CM- I33.0, I39, I33.9, I38, A3282, B376) based on supplemental tables from Schranz et al. and Nenninger et al.^3^,^13^ and by manually examining the code lists. Those codes have been validated and used in prior studies to identify IE hospitalizations with 94% and 90% sensitivities and positive predictive values of 94% and 80% for ICD 9 and 10 codes, respectively.^14^,^15^

The IDU-IE admissions were identified using additional ICD diagnosis codes for injected illicit drug use, including opioids, cocaine, amphetamines, benzodiazepines, and hallucinogens (Supplemental Table S2). Diagnosis codes that included use, dependence, poisoning, or withdrawal were considered IDU-related IE cases. For individuals born after 1965, a diagnosis of hepatitis C virus (HCV) was also considered a marker of drug use, given the compelling evidence linking HCV infection to intravenous drug use. 4,15,16 All remaining IE admissions not meeting these criteria were considered non-IDU-IE.

### Statistical Analysis

#### Descriptive Statistics

Descriptive statistics were reported as counts and percentages for categorical variables. Statistical significance was assessed at *p* ≤ 0.05. The Pearson Chi-square test was used to compare characteristics between IDU-IE and non-IDU-IE groups. Data analyses were performed using SAS version 9.4 statistical software (SAS Institute Inc, Cary, NC).

KY population data by sex, age, and county were obtained from the U.S. Census Bureau.^17^ Annual hospitalization rates for (IE) per 100,000 KY residents at both state and county levels were calculated by dividing the yearly number of IE admissions by the KY Census population for that same year and the corresponding county population, respectively. The Appalachian counties were defined according to the Appalachian Regional Commission. Joinpoint regression analysis was used to identify periods with changes in trends and estimate annual percent changes (APC) via the Joinpoint regression software (version 4.9.1.0).^18^

#### Spatiotemporal Cluster Detection Analysis

A spatiotemporal cluster analysis was conducted using SaTScan (version 9.6.0.0), a statistical software used for outbreak surveillance in infectious disease epidemiology^19^–^21^ and identifying high-risk areas in cancer epidemiology.^22^,^23^ The software uses cylindrical windows to identify clusters, where the bottom circle represents the scanning area and the height represents the scanning time. The analysis was performed on a county level, and we used a 10-mile grid of points instead of county centroids as the basis for candidate clusters to balance spatial resolution with statistical stability and computational efficiency across the statewide analysis. The high-rate clusters were identified using discrete Poisson modeling, adjusted for age and sex. The primary analysis maximum spatial cluster size was initially set to 50% of the population at risk, which identified two large clusters that were not ideal for identifying possible counties eligible for intervention. The reported analysis maximum spatial cluster size was then set at 25% of the population at risk. The authors chose not to set the population at risk less than 25% to keep the identified cluster from Louisville. For inference, we used 999 Monte Carlo replications. Maps were generated using QGIS 3.4.15 to visualize clusters across KY counties.

## RESULTS

### Characteristics of Hospitalizations by Drug Use Status

**Figure 1:**
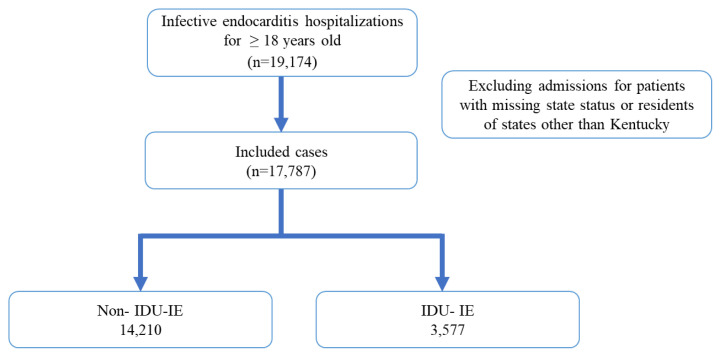
Study population diagram

Of the 19,174 IE hospitalizations among adults 18 years of age and older between 2008–2018, 17,787 were for residents of the state (Figure 1). A total of 14,210 hospitalizations were non-IDU-IE, and 3,577 were IDU-IE. The majority of IDU-IE admissions (2,899 out of 3,577; 81%) were for patients between 18 and 45 years old, while 7,307 out of 14,210; 51% of patients hospitalized for non-IDU-IE were 65 years and older (Table 1). IDU-IE hospitalizations were slightly more common among male patients (52%) than females (48%). The percentage of white individuals among IDU-IE was 94%. Significantly more Medicaid patients (75% versus 15%) were in the IDU-IE group compared to the non-IDU-IE group.

Each substance type was assessed independently because a single hospitalization could involve more than one drug. Among the 3,577 drug-related hospitalizations, 2,417 (67.6%) involved opioids, 702 (19.6%) involved amphetamines, 344 (9.6%) involved cocaine, 14 (0.4%) involved benzodiazepines or hallucinogens, and 2,039 hospitalizations (57%) had documented hepatitis C infection among individuals born after 1965. These categories are not mutually exclusive, and totals may exceed 100% due to poly–drug use.

### Annual Trends in IE Hospitalizations

**Figure 2:**
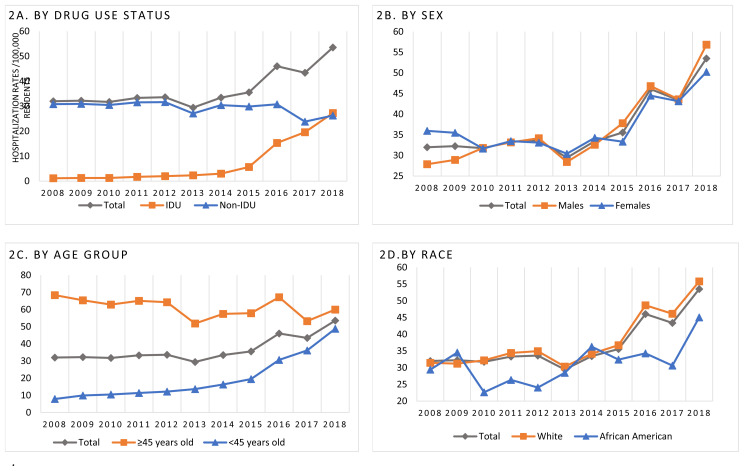
Trends of IE hospitalization rates in Kentucky (2008-2018) *The denominators in graphs 2B, 2C & 2D are within the stratum*.

The total number of IE admissions in KY increased from 1,372 in 2008 to 2,388 in 2018. Annual hospitalizations for IDU-IE rose from 50 in the state in 2008 (1.2 per 100,000) to 1216 (27.3 per 100,000) in 2018, an approximately 23-fold increase (Figure 2A). The joinpoint regression identified a period between 2013 and 2018 with a significant increase in the trend, with an estimated APC for IDU-IE hospitalizations during that timeframe reaching 71.3% (95% CI: 47.8,98.5). Yearly hospitalization rates for non-IDU-IE ranged between 23.8 and 31.6 per 100,000 for the study period, and the joinpoint regression identified a decreasing trend between 2008 and 2018 with an APC of −1.8% (95% CI: −3.3, −0.2). The observed annual IE hospitalizations trends are roughly similar for men and women (Figure 2B).

Figure 2C shows that the observed increase in IE hospitalizations was most pronounced among those 18 to 45 years old. Joinpoint regression identified a significant increase between 2014 and 2018, with an estimated APC of 33.4% (95% CI: 24.3,43.1). For those with IDU-IE, a large increase was noted specifically in the 26–35 age group (Supplemental Figure S1).

Hospitalization rates among African Americans increased between 2010 – 2018 with an estimated APC of 6.2% (95% CI: 1.2,11.5), with 22.6 admissions per 100,000 African Americans IE hospitalizations in 2010 versus 45 per 100,000 in 2018. Hospitalization rates among whites increased during the years 2014 – 2018, with an APC of 13.4% (95% CI: 4.4,23.1) (Figure 2D).

The geospatial distribution of IE and IDU-IE hospitalization rates by county shows an increase in IDU-IE admissions in 2018 compared to 2008, mainly in Eastern KY (Figure 3A & 3B). Other/unknown 666 (4) 530 (4) 136 (4)

### Cluster analysis

**Figure 4:**
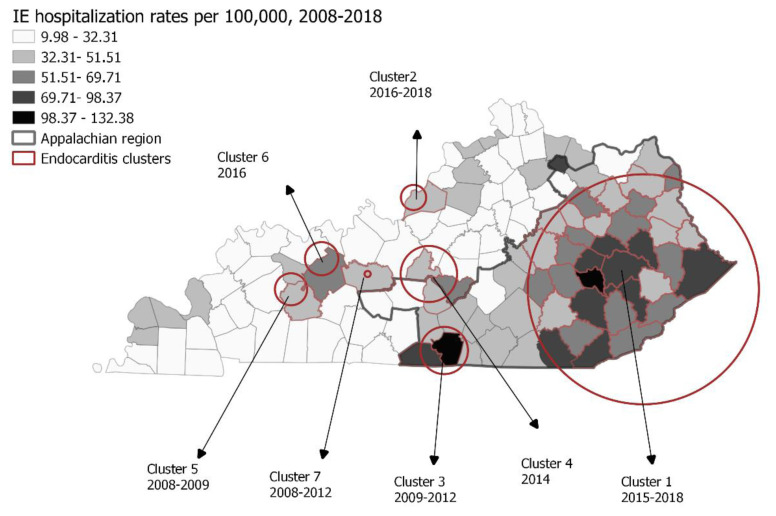
Choropleth map of average rates of county-level IE hospitalization rates and high-rate clusters

We identified seven statistically significant clusters of IE hospitalizations throughout the study period (Figure 4). Cluster 1, comprising 2379 IE hospitalizations, occurred between 2015 – 2018 and involved 34 counties in Eastern KY (identified by name in Table 2). The relative risk of IE hospitalization in this cluster was 2.06, *p* <0.0001 compared with the rest of the state during the same time period. The second largest, cluster 2, included 1616 IE hospitalizations identified in Jefferson County between 2016 – 2018. This cluster’s relative risk for IE hospitalization rates was 1.98, *p* <0.0001. The other most recent clusters were cluster 4, which included Larue, Green, and Taylor Counties in 2014, with a relative risk of 3.13, *p* =0.0001, and cluster 6 in Ohio County in 2016, with a relative risk of 3.24, *p* =0.0038.

## DISCUSSION

We observed an increase in IE admissions occurring in KY during the years 2008 – 2018. This increase was associated with hospital admissions for IDU-IE, which were most common among patients 26–35 years of age. The IDU-IE hospitalization rates increased from 1.2 per 100,000 KY residents in 2008 to 27.3 per 100,000 in 2018, with an estimated APC of 71.3% (95% CI: 47.8,98.5) from 2013 – 2018. One critical finding of this study was identifying a prominent cluster of 2379 IDU-IE hospitalizations in Eastern KY between 2015 – 2018.

Our findings are comparable to results from studies conducted in NC, where IDU-IE hospitalization rates increased approximately 12-fold from 2007 to 2017 (0.92 to 10.95 per 100,000 NC residents).^3^ Notable increases were also reported for IDU-IE hospitalizations in Virginia (7.5-fold increase from 2000 to 2016),^24^ West Virginia (3-fold increase from 2014 to 2018),^25^ and Oregon (8-fold rise from 2008–2018).^26^ Rates of IE hospitalizations observed in this study align with national rates reported in two studies using national inpatient sample data and documenting increases in IDU-IE.^13^,^27^ The disproportionate increase in IDU-IE hospitalizations among individuals aged 26–35 years is consistent with national mortality and surveillance data demonstrating the highest burden of injection drug use–related harms in this age group.^28^ This pattern may reflect a cohort effect characterized by earlier exposure to opioids, progression to injection drug use,^29^ and cumulative risk of infectious complications by early to mid-adulthood, resulting in greater clinical severity and hospitalization compared with both younger and older populations.

The spatiotemporal analysis showed that IE hospitalizations disproportionately affected the Appalachian region of KY during the years 2015–2018. The overall increase in IE in this particular region is mainly attributable to IDU, mirroring the opioid use crisis in rural Appalachia.^30^ This geographic disparity coincides with the prevalence of risk factors associated with opioid use in the region, including poverty and unemployment.^31^ Based on data from the American community survey from 2011 – 2015, the Appalachian region of KY has a high percentage of the population living in poverty, with several counties in that region, including Wolfe, Bell, Knox, and Breathitt, among the highest rates of poverty in the nation, ranging from 33% – 43%.^32^ Similarly, in 2015, the unemployment rates in counties in the identified clusters (except cluster 2) were higher than in the rest of the state.^33^ Prior literature suggests that community-level strategies such as expanded access to medication for opioid use disorder,^34^ integration of addiction and infectious disease care,^35^ as well as taking a multidisciplinary addiction care team approach, particularly in rural and high-risk regions, are associated with reductions in injection-related harms and infectious complications.^36^,^37^

### Limitations

This study has several limitations. Administrative hospitalization data used in the study could have included coding errors and subsequent event misclassification. The switch from ICD-9-CM codes to ICD-10-CM in 2015 may have introduced errors in trend estimates. Furthermore, the data were limited to hospitalizations occurring in KY only; therefore, information for patients transferred and treated in neighboring states were not captured, and IE hospitalizations in border counties may have been underestimated. Additionally, hospital discharge data included only IE patients admitted to a hospital, excluding those with severe symptoms who died before visiting the Emergency Room or less severe cases not receiving in-hospital medical attention. The data also do not include Veterans Administration and military hospital discharges. Lack of personal identifiers precluded estimation of readmission rates or the prevalence of IE. For the same reason, interfacility transfers within KY may be counted as separate hospitalizations, which could lead to overestimation of total hospitalization counts. Another limitation involves the exposure assessment for IDU-IE, with the data lacking specifics on drug doses and misuse duration. Despite those limitations, our findings emphasize the urgent need for community interventions to prevent IDU and support patients’ long-term recovery from opioid misuse, especially in the identified geographic clusters.

## CONCLUSION

Study findings strongly emphasize the magnitude of the opioid epidemic in the Appalachian region of KY. Admissions for IE represent an opportunity for addiction treatment interventions in people who inject drugs. Future research evaluating clinical outcomes and management of IDU-IE in hotspots may provide additional insights into ways to decrease the burden of this disease in the state.

**SUMMARY BOX**

**What is already known about this topic?**

Infective endocarditis (IE) hospitalization rates have increased in the United States over the past decade, largely driven by the opioid epidemic and rising intravenous drug use (IDU). However, there is limited evidence describing state-and county-level spatial and temporal trends, particularly in high-burden states such as Kentucky.

**What is added by this report?**

This study quantifies the rapid rise in IDU-associated infective endocarditis (IDU-IE) hospitalizations in Kentucky, showing an increase from 1.2 to 27.3 per 100,000 population between 2008 and 2018, with a significant acceleration from 2013–2018 (annual percent change [APC]: 71.3%). The study also identifies seven significant county-level spatiotemporal clusters of elevated IE risk, with the largest cluster in Eastern Kentucky demonstrating more than a twofold increased risk (RR=2.06).

**What are the implications for future research?**

These findings underscore the need for targeted, geographically informed public health interventions to address IDU-related IE in high-risk areas. Future research should explore the underlying drivers of these spatial disparities, evaluate prevention strategies, and assess the healthcare burden associated with IDU-related IE at both local and national levels.

## Supplementary Information



## Figures and Tables

**Figure 3A:**
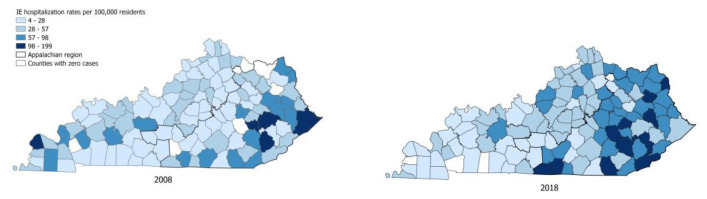
Choropleth maps of county-level IE hospitalization rates per 100,000 residents

**Figure 3B:**
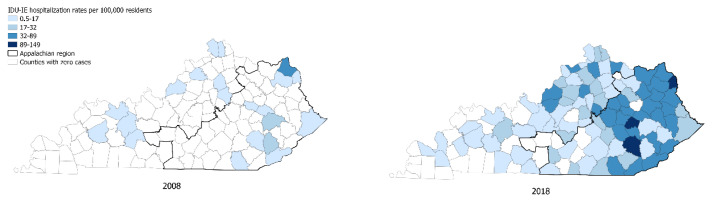
Choropleth maps of county-level IDU-IE hospitalization rates per 100,000 residents

**Table 1:** IE hospitalizations by IDU status-Hospital inpatient discharges data, KY (2008–2018)

	Total N=17,787	Non-IDU-IE N= 14,210 (%)	IDU-IE N= 3577 (%)	*p*-value
**Age**				<0.0001
18–25	869 (5)	385 (3)	484 (14)	
26–35	2525 (14)	1053 (7)	1472 (41)	
36–45	2168 (12)	1225 (9)	943 (26)	
46–55	2178 (12)	1748 (12)	430 (12)	
56–65	2692 (15)	2492(18)	200 (6)	
65+	7355 (41)	7307 (51)	48(1)	
**Sex**				0.0007
Male	8733 (49)	6886 (48)	1847 (52)	
Female	9054 (51)	7324 (52)	1730 (48)	
**Race**				<0.0001
White	16,179 (91)	12,816 (90)	3363 (94)	
African American	1230 (7)	1066 (8)	164 (5)	
Others	378 (2)	328 (2)	50 (1)	
**Primary source payer**				<0.0001
Medicare	9341 (53)	8984 (63)	357 (10)	
Medicaid	4843 (27)	2174 (15)	2669 (75)	
Private	2447 (14)	2178 (15)	269 (8)	
Self-pay	490 (3)	344 (2)	146 (4)	
Other/unknown	666 (4)	530 (4)	136 (4)	

NOTE: p-value obtained from chi-square test comparing IDU-IE to non-IDU-IE

**Table 2:** Statistically significant spatiotemporal clusters of IE in Kentucky (2008–2018) adjusted for age and sex

	County	Time period	No. of observed cases	No. of expected cases	hospitalization rates/per 100,000	RR	*p*-value
Cluster 1	Knott, Perry, Letcher, Breathitt, Leslie, Floyd, Magoffin, Harlan, Owsley, Clay, Wolfe, Pike, Johnson, Lee, Morgan, Martin, Jackson, Knox, Menifee, Bell, Powell, Estill, Lawrence, Elliott, Laurel, Rowan, Montgomery, Bath, Rockcastle, Carter, Whitley, Madison, Clark, Boyd	2015–2018	2379	1238.6	87.4	2.06	<0.0001
Cluster 2	Jefferson	2016–2018	1616	852.9	86.3	1.98	<0.0001
Cluster 3	Cumberland, Monroe	2009–2012	80	27.5	132.4	2.92	<0.0001
Cluster 4	Larue, Green, Taylor	2014	60	19.2	142.0	3.13	0.0001
Cluster 5	Muhlenberg	2008–2009	59	24.0	111.9	2.46	0.0001
Cluster 6	Ohio	2016	29	8.9	147.5	3.24	0.0038
Cluster 7	Grayson	2008–2012	86	48.0	81.5	1.79	0.020
