# Supplementary material for: Infective Endocarditis Hospitalizations in Kentucky, 2008–2018: Spatial and Temporal Trends
Source: J Appalach Health. 2026 Apr 1;8(1):22–41. doi: 10.13023/jah.0801.03 (PMC13233083; doi:10.13023/jah.0801.03)
Supplement: Supplementary file 1 [file JAH_8.1.3_Additionalfile.docx]

Figure S1. Trends of intravenous drug use infective endocarditis by age group.

| Table S1. ICD codes for infective endocarditis diagnosis | | | |
| --- | --- | --- | --- |
| **ICD-9** | | **ICD-10** | |
| 4210 | Acute and subacute bacterial endocarditis | I33.0 | Acute and subacute endocarditis |
| 4211 | Acute and subacute infective endocarditis in diseases classified elsewhere | I33.9 | Acute and subacute endocarditis, unspecified |
| 4219 | Acute endocarditis, unspecified | I39 | Endocarditis and heart valve disorders in diseases classified elsewhere |
| 42490 | Endocarditis, valve unspecified, unspecified cause | I38 | Endocarditis, valve unspecified |
| 42491 | Endocarditis in diseases classified elsewhere | A3282 | Listerial endocarditis |
| 42499 | Other endocarditis, valve unspecified | B376 | Candidal endocarditis |
| 11281 | Candida endocarditis |  |  |

| Table S2. ICD codes for drug use and related markers | | |
| --- | --- | --- |
|  | ICD-9 | ICD-10 |
| Opioids | '30400', '30401', '30402', '30403','30470', '30471', '30472', '30550',  '30551', '30552', '96500', '96501','96502', '96509', 'E8500', 'E8501', 'E8502', 'E9350', 'E9351', 'E9352' | 'F1110', 'F11120', 'F11121', 'F11122', 'F11129', 'F1114', 'F11150','F11151', 'F11159', 'F11181', 'F11182','F11188', 'F1119', 'F1120', 'F11220',  'F11221', 'F11222', 'F11229', 'F1123', 'F1124', 'F11250', 'F11251', 'F11259', 'F11259', 'F11281', 'F11282', 'F11288', 'F1129', 'F1190', 'F11920', 'F11921', 'F11922', 'F11929', 'F1193', 'F1194', 'F11950', 'F11951', 'F11959', 'F11981', 'F11982', 'F11988', 'F1199''T400X5A', 'T400X5D', 'T400X5S', 'T402X5A', 'T402X5D', 'T402X5S', 'T403X5A', 'T403X5D', 'T403X5S', T404X5A', 'T404X5D', 'T404X5S', 'T40605A', 'T40605D', 'T40605S', 'T40695A', 'T40695D', 'T40695S', 'T400X1A', 'T400X1D', 'T400X1S', 'T400X4A', 'T400X4D', T400X4S', 'T401X1A', 'T401X1D', 'T401X1S', 'T401X4A', 'T401X4D', 'T401X4S', 'T402X1A', 'T402X1D', 'T402X1S', 'T402X4A', 'T402X4D', 'T402X4S', T403X1A', T403X1D', 'T403X1S', 'T403X4A', 'T403X4D', 'T403X4S', 'T404X1A', 'T404X1D', 'T404X1S', 'T404X4A', 'T404X4D', 'T404X4S', 'T40601A', 'T40601D', 'T40601S', 'T40604A', 'T40604D', 'T40604S', 'T40691A', 'T40691D', 'T40691S', 'T40694A', 'T40694D', 'T40694S' |
| Benzodiazepines | '9670', '9694', 'E851', 'E8532',  'E9501', 'E9801', '9670', '9694' | 'T423X1A','T423X1D','T423X1S','T423X4A','T423X4D'  'T423X4S','T424X2A','T424X2D','T424X2S','T423X1A',  'T423X1D' |
| Cocaine | '30420', '30421', '30422', '30560','30561', '30562', '97081', 'E9385' | 'F1410','F14120','F14121','F14122','F14129',  'F1414','F14150','F14151','F14159','F14180',  'F14181','F14182','F14188','F1419','F1420','F14220',  'F14221','F14222','F14229','F1423','F1424','F14250',  'F14251','F14259','F14280','F14281','F14282',  'F14288','F1429','F1490','F14920','F14921',  'F14922','F14929','F1494','F14950','F14951',  'F14959','F14980','F14981',  'F14982','F14988','F1499' |
| Methamphetamines or Stimulants | '3044', '3057', '96972', '96973',  '96979', '9709', 'E8542', 'E8543' | 'F1510','F15120','F15121','F15122','F15129',  'F1514','F15150','F15151','F15159','F15180',  'F15181','F15182','F15188','F1519','F1520',  'F15220','F15221','F15222','F15229','F1523',  'F1524','F15250','F15251','F15259','F15280',  'F15281','F15282','F15288','F1529','F1590',  'F15920','F15921','F15922','F15929','F1593',  'F1594','F15950','F15951','F15959','F15980',  'F15981','F15982','F15988','F1599','T43622A',  'T43622D','T43622S','T43632A','T43632D',  'T43632S','T43692A','T43692D','T43692S','T43605A','T43605D','T43605S' |
| Hepatitis C | '7041', '7044', '7051', '7054', '7070', '7071', 'V0262' | 'B1710','B1711','B182','B1920','B1921','Z2252' |
| Hallucinogens | '3045', '3053', '9696', 'E8541', 'E9396' | 'F1610','F16120','F16121','F16122','F16129','F1614',  'F16150','F16151','F16159','F16180','F16183',  'F16188','F1619','F1620','F16220','F16221','F16229',  'F1624','F16250','F16251','F16259','F16280',  'F16283','F16288','F1629','F1690','F16920',  'F16921','F16929','F1694','F16950','F16951',  'F16959','F16980','F16983','F16988','F1699',  'T408X1A','T408X1D','T408X1S','T408X4A',  'T408X4D','T408X4S','T40901A','T40901D',  'T40901S','T40904A','T40904D','T40904S',  'T40991A','T40991D','T40991S','T40994A',  'T40994D','T40994S' |
